# Long-term survey of sea turtles (*Caretta caretta*) reveals correlations between parasite infection, feeding ecology, reproductive success and population dynamics

**DOI:** 10.1038/s41598-020-75498-4

**Published:** 2020-10-29

**Authors:** Emma C. Lockley, Leila Fouda, Sandra M. Correia, Albert Taxonera, Liam N. Nash, Kirsten Fairweather, Thomas Reischig, Jandira Durão, Herculano Dinis, Silvana Monteiro Roque, João Pina Lomba, Leno dos Passos, Sahmorie J. K. Cameron, Victor A. Stiebens, Christophe Eizaguirre

**Affiliations:** 1 School of Biological and Chemical Sciences, Queen Mary University of London, Mile End Road, London, E14NS UK; 2 Instituto Do Mar I.P. (IMar), Cova de Inglesa, C.P 132, Mindelo, Ilha do São Vicente, Cabo Verde; 3 Associação Projeto Biodiversidade, Mercado Municipal 22, Santa Maria 4111, Ilha do Sal, Cabo Verde; 4 Turtle Foundation, An der Eiche 7a, 50678 Cologne, Germany; 5 Biosfera I, Rua de Moçambique 28, Mindelo, Ilha do São Vicente, Cabo Verde; 6 Associação Projecto Vitó, Xaguate, São Felipe, Ilha do Fogo, Cabo Verde; 7 Projeto Vitó Porto Novo, Porto Novo, Ilha do Santo Antão, Cabo Verde; 8 Associação Ambiental Caretta Caretta, Achada Igreja, Pedra Badejo, Santa Cruz, Ilha do Santiago, Cabo Verde; 9 Fundação Maio Biodiversidade, Cidade de Porto Inglês, Ilha do Maio, Cabo Verde

**Keywords:** Conservation biology, Evolutionary ecology, Population dynamics

## Abstract

Long-term monitoring of host-parasite interactions is important for understanding the consequences of infection on host fitness and population dynamics. In an eight-year survey of the loggerhead sea turtle (*Caretta caretta*) population nesting in Cabo Verde, we determined the spatiotemporal variation of *Ozobranchus margoi*, a sanguivorous leech best known as a vector for sea turtle fibropapilloma virus. We quantified *O. margoi* association with turtles’ δ^15^N and δ^13^C stable isotopes to identify where infection occurs. We then measured the influence of infection on reproduction and offspring fitness. We found that parasite prevalence has increased from 10% of the population in 2010, to 33% in 2017. Stable isotope analysis of host skin samples suggests transmission occurs within the host’s feeding grounds. Interestingly, we found a significant interaction between individual size and infection on the reproductive success of turtles. Specifically, small, infected females produced fewer offspring of poorer condition, while in contrast, large, infected turtles produced greater clutch sizes and larger offspring. We interpret this interaction as evidence, upon infection, for a size-dependent shift in reproductive strategy from bet hedging to terminal investment, altering population dynamics. This link between infection and reproduction underscores the importance of using long-term monitoring to quantify the impact of disease dynamics over time.

## Introduction

Host-parasite interactions are sensitive to environmental changes^1–3^. Long-term monitoring of these interactions can serve as an early warning signal before major ecosystem shifts occur. For instance, a nine year survey of avian malaria in blue tits (*Cyanistes caeruleus)* revealed oscillations in transmission among years that were driven by temporal fluctuations of vector abundance^4^. In this system, acute infection caused significant blue tit mortality^5^. In another long-term study, parasite infection resulted in selection against inbreeding within a small population of Soay sheep (*Ovis aries*), as infected individuals with low genome-wide heterozygosity had lower fitness^6^. While the patterns of disease transmission and virulence in response to environmental perturbations remain difficult to predict (as disease transmission may be elevated^7^ or reduced^8^), long-term studies can facilitate clarifying modes of parasite transmission, and the effects of infection on critical life history traits such as feeding and reproduction. This information is particularly valuable when host populations are already at risk of extinction.

Deterministic models suggest that in the vast majority of host-parasite systems, parasites should become extinct before their hosts. This is because at low density the probability for parasites to find a host and survive is reduced. Furthermore, in some wild populations, the presence of parasites can drive the evolution of strategies that can compensate for the costs of infection and reduce negative effects on host populations^9^. There are, however, exceptions to those outcomes of infection: frequency-dependent transmission, stochastic extinction at low densities, and biotic or abiotic reservoirs for parasites may all drive vulnerable host populations to extinction^10^. A notorious example is that of the pathogenic chytrid fungus, *Batrachochytrium dendrobatidis*, responsible for the decline of 6.5% of amphibian species and at least 90 presumed extinctions worldwide^11^. The fungus’ ecological success is linked to increasingly favourable environmental conditions resulting from climate change^11^. Although direct mortality is of particular concern in the case of the chytrid fungus, indirect effects of infection on the host, from foraging behaviours through to reproductive output, may also have significant effects on host population dynamics^12,13^, and determining the direction of these effects requires thorough investigations.

An efficient feeding strategy is arguably one of the most important life history traits of an individual, regulating nutrient uptake, physiology and reproduction^14,15^. Feeding rate is commonly influenced by infection, with hosts often increasing levels of food consumption to meet the energetic demand of mounting an immune response^16,17^. Alternatively, in some cases feeding rate can be reduced: when infected by common parasites, three-spined stickleback fish, *Gasterosteus aculeatus*, demonstrate a preference for smaller prey, resulting in the assimilation of less nutritious items, presumably to reduce competition with uninfected fish^18^. In extreme situations, parasites and pathogens may prevent feeding of their host entirely, as in the case of sea turtle fibropapillomatosis, which leads to cutaneous lesions on soft tissues that impair vision, locomotion and eating capacity before death^19,20^. In these cases, the impact of infection may be detected by a change in a host’s trophic niche as an early warning signal before severe symptoms appear^21,22^.

In natural populations, causal relationships between infection and ecological niche shift are difficult to ascertain. Infection can lead to host niche shifts, but niche-use may alternatively influence a potential host’s exposure to certain parasites^22^. Because exposure to parasite communities can vary greatly across trophic niches, it is possible to detect the spatial distribution of parasites, and the risks of infection when trophic niches are segregated within a population^23^. Through the use of stable isotopes—a continuous measure of energy flow through trophic levels and communities—it is possible to estimate trophic niches of individuals. In particular, the ratio of nitrogen stable isotopes (δ^15^N) of a consumer is normally enriched by 3–4 ‰ in comparison to their prey^24^. Carbon ratios (δ^13^C) vary much less throughout a trophic web (approximately 1 ‰), and instead provide information on the original source of the carbon, thus revealing the foraging habitat of an organism^24^. In marine ecosystems, a more depleted δ^13^C value indicates the use of oceanic foraging areas, while the δ^13^C values of coastal foragers are higher^24^. With stable isotope analysis, it is therefore possible to (1) determine habitats where risks of infection are highest, and (2) explore the link between foraging ecology and parasite burden.

The combined effects of infection and feeding ecology can also influence resource allocation trade-offs between life history traits, such as those associated with reproduction and survival^25,26^. In response to infection, reproductive output can be either reduced^27,28^ or increased^29–31^, broadly depending on whether bet-hedging or terminal investment strategies are adopted. Reduced host fecundity can be a direct consequence of resource exploitation by parasites, but alternatively might be indicative of resource reallocation from current reproduction to survival (and future reproduction) until the infection has passed—a bet-hedging strategy^32^. For instance, triggering an artificial immune response in the lizard *Ctenophorus fordi* reduces host reproductive investment as quantified by egg mass, because a trade-off exists between current and future reproductive events^33^. Alternatively, in situations where recovery is unlikely, strategic terminal investment should instead be favoured, resulting in higher reproductive performance in infected individuals during their final reproductive attempts^29–31^. These strategies can even co-exist in species depending on life stages. In the blue-footed booby, *Sula nebouxii*, the reproductive success of individuals mounting an immune response is lower in young males, whereas older males nearing senescence show an increase in breeding success as high as 98%^34^. Finally, responses to infection may span generations via mechanisms known as trans-generational immune priming, which can be advantageous to host progeny if they share the same pathogenic environment as their parents^35–37^.

Here, using data from eight years of intensive monitoring of nesting female loggerhead sea turtles, *Caretta caretta*, in Cabo Verde, we tested (1) whether the prevalence of a leech infection changes over time, (2) where infection occurs, (3) whether infection relates to host foraging strategy, and (4) whether it impacts the demography of this endangered population. Specifically, we used stable isotope analysis to investigate the spatiotemporal occurrence of a sea turtle specific leech ectoparasite, *Ozobranchus* sp., and to relate it to trophic niche and reproductive investment. Little is known about the life-cycle of this leech, although, as all stages of development have been recorded on their host, it is possible that they complete their entire life cycle on turtles^38^. Sea turtle superinfection (defined as > 1000 leeches) by the parasite can cause erosion of soft tissues and bone, and can lead to death^39,40^. In addition, *Ozobranchus* sp. are also the most likely vector of the chelonid herpesvirus ChHV5, which is associated with sea turtle fibropapillomatosis, a potentially fatal neoplastic condition of sea turtle species^41,42^. The role of *Ozobranchus* sp. as a vector of turtle fibropapillomatosis is of high conservation concern, but little is known about the direct effects of infection by the leech itself on feeding ecology or reproduction in natural populations. As sea turtle fibropapillomatosis has not yet been reported in loggerhead turtles in Cabo Verde, it makes this population ideal to consider the effects of this parasite independently of this co-infection. Composed of several philopatric nesting groups, the Cabo Verde population is the third largest loggerhead sea turtle population in the world and the only significant one in the eastern Atlantic, making its conservation not only of local but also of global concern^43,44^.

## Methods

All methods adhered to relevant guidelines and regulations outlined by Queen Mary University of London for the care and use of animals. All sample collection and experiments adhered to national legislation, and were approved by the Direção Nacional do Ambiente of Cabo Verde (authorisations: DGA 30/13, DGA 27/2014, DNA 30/2015, DNA 25/2016, DNA 46/2017, DNA 67/2018).

### Spatiotemporal trends in parasite prevalence

**Figure 1:**
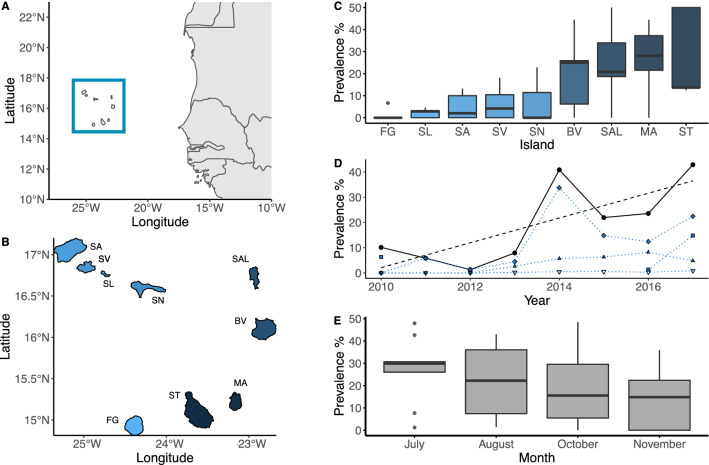
Spatiotemporal variation of *O. margoi* infection. (**A**) The Cabo Verde archipelago is located of the west coast of Africa. Map created using R package “rnaturalearth” v 0.1.0 (https://CRAN.R-project.org/package=rnaturalearth) and “ggplot2” v3.3.2 (Wickham 2016, https://ggplot2.tidyverse.org). (**B**) Map of the islands of Cabo Verde, with colour showing average parasite prevalence reported in C (SA = Santo Antão, SV = São Vicente, SL = Santa Luzia, SN = São Nicolao, SAL = Sal, BV = Boa Vista, MA = Maio, ST = Santiago, FG = Fogo). Shape files hand drawn from Google Earth, (Data SIO, NOAA, U.S. Navy, NGA, GEBCO https://earth.google.com/web/). (**C**) Boxplot showing the parasite prevalence on the nine islands where sampling was conducted. (**D**) Overall significant increase in parasite prevalence between 2010 and 2017 (black), along with island-specific parasite prevalence for the eastern islands (Χ^2^ = 38.357, df = 8, *p* < 0.001). Diamond = Boa Vista, solid triangle = Maio, square = Sal and empty triangle = Santiago. (**E**) Boxplot showing the monthly parasite prevalence over the nesting season. Note, the analysis used month as a continuous variable to consider the auto-correlated nature of this temporal variable (Χ^2^ = 5.501, df = 1, *p* = 0.019).

During the months of July to October in 2010 to 2017, nesting female turtles were sampled from across the Cabo Verde archipelago (N _turtles_ = 4386), from a total of nine islands (Fig. 1A,B). After oviposition, turtles were tagged with a PIT (AVID) and/or metal (Inconel) tag to allow for identification during subsequent nesting events^44^. Notch-to-notch curved carapace length (CCL) was measured (± 0.1 cm), and the presence or absence of *Ozobranchus* sp. on the cloaca of individuals was recorded. While all soft tissues of turtles are checked for parasites, in this study, leeches were found only around the cloaca and therefore we assume the cloacal presence to be an accurate representation of the infection status. Abundance could not be unambiguously determined, but no turtle had more than 50 leeches. Samples of the leech were collected in order to confirm species identity. A 3 mm sample of non-keratinised tissue was taken from the front flipper of each turtle for stable isotope analysis^48^. These data were used to quantify an overall spatiotemporal trend in parasite prevalence across the archipelago. In 2018, a further 88 turtles were sampled in this manner on the island of Sal to directly test the impact of infection on reproduction. The raw data are available from TurtleBase (www.qmul.ac.uk/eizaguirrelab/turtlebase), as part of a citizen-science project that protects sea turtles in Cabo Verde^45^.

### DNA extraction and species confirmation

In order to identify the leech species infecting turtles, we extracted DNA from 90 leeches randomly selected over space and time using the DNeasy 96 Blood and Tissue kit (Qiagen, Hilden, Germany), according to the manufacturer’s protocol. We then amplified 654 bp of the Nicotinamide adenine dinucleotide dehydrogenase subunit 1, NADH as well as 600 bp of the 18S small subunit ribosomal DNA gene. We used primers LND300 (TGGCAGAGTAGTGCATTAGG) and HND1932 (CCTCAGCAAAATCAAATGG^46^) for NADH and the modified primers from^47^ for the 18S rDNA. PCR reactions and thermo-cycling protocols can be found in supplementary material (Supplementary Table S1).

### Infection and turtle foraging strategy

To determine δ^15^N and δ^13^C isotope ratios in adult female turtles, tissue samples from a random sub-sample (n = 926) of the population were washed in distilled water to remove contamination from sand, and dried for 48 h at 60 °C^48^. Between 0.7 and 1.3 μg of ground sample were measured into 4 mm tin capsules. A continuous flow isotope ratio mass spectrometer (Integra2, Sercon) combusted the samples, and concurrently quantified δ^15^N and δ^13^C. Only samples collected from an individual’s first recorded nesting event of the season were included in order to maintain an accurate representation of foraging strategy prior to the nesting migration^48^.

### Infection and reproductive success

The clutches of a subset of females that had been sampled for stable isotope analysis (n = 244) on two islands were relocated to in situ experimental hatcheries immediately after oviposition. The hatcheries were outdoor enclosures located on the beach that protected nests from tidal inundation and predation, while still allowing them to be exposed to natural environmental conditions. These relocation studies occurred in 2016 and 2017 on the island of Boa Vista (n = 20 and 39 respectively), and 2017 and 2018 on the island of Sal (n = 97 and 88 respectively). The number of eggs per clutch was recorded, and for 126 clutches the mass and diameter of two randomly selected eggs were also measured. Clutch mass was calculated as a product of the average weight of these two eggs and the number of eggs in the clutch. Nest success was calculated as the percentage of eggs that resulted in a successfully emerged hatchling.

### Trans-generational effect of infection

The incubation duration of nests was defined as the number of days between oviposition and hatchling emergence. Upon emergence, between 20 and 25 hatchlings from each nest (N_nest_ = 244, N_offspring_ = 5048) were randomly selected for fitness trait measurements. In this population, hatchling size correlates with swimming performance^49^, and so the hatchlings were weighed (± 0.1 g), and the notch-to-notch straight carapace length (SCL) was measured using digital callipers (± 0.01 mm). Two further fitness traits involved in natural predator avoidance during hatchling emergence, crawl speed and time to self-righting, were determined for offspring from 186 of these nests (Crawl Speed N_offspring_ = 3671, Self-righting time N_offspring_ = 3897). Crawl speed was measured by recording the time for an individual to crawl the length of a 0.5 m piece of PVC guttering lined with sand, with a dull red light placed at one end of the runway. This trial was repeated twice, and an average was taken (cm/s). Self-righting capacity was measured by placing a hatchling on its back on an area of flat sand and recording the time to right itself^50^. This trial was repeated three times per individual, and if the hatchling took longer than 60 s to self-right it was considered to have failed the trial. We measured both the number of successful trials (0–3) and the average self-righting time (using successful events, in seconds). All hatchling data can be found in Supplementary Data File 1.

### Statistical analyses

Statistical analyses were conducted in R version 3.3.3. All models were backward selected using AIC values to retain the optimal reduced model. Where there were colinearities between fixed variables, we replaced one of these variables with the residuals of its regression against the second (Supplementary Table S2). The statistical approach implemented linear and generalised mixed effect models such that intercept-only parameters could be fitted to account for non-independence in our response variables where known groupings in the data might account for some of the variation. For example, where individuals from the same nest were sampled, maternal ID was included as a random (intercept only) effect to account for this non-independence.

To test whether parasite prevalence varied across the archipelago and over time, parasite presence/absence was fitted in a binomial generalised linear model (GLM) with year, island and turtle size (CCL) as predictors, along with all two-way interactions. To determine the seasonal trend within the sampling years, a binomial generalised linear mixed effect model (GLMM) that included month, island, CCL and their interactions as fixed predictors was used, with year included as a random effect.

Linear mixed effect models (LMM) were used to determine the relationship between infection and feeding ecology. Two separate models, for δ^15^N and δ^13^C respectively, were conducted with parasite presence/absence, CCL and their interactions as fixed predictors. As differences in feeding strategy have been observed among islands, we included island as a random factor, along with year^48^.

The effect of infection on reproductive parameters (egg mass, egg density, clutch mass and clutch size) was established using independent LMMs that included year and island as random effects, and parasite presence/absence along with δ^15^N and δ^13^C, as well as their interactions as fixed variables. As CCL is a well-known correlate of clutch size and reproductive investment in turtles, it was included as a covariate^51^. The nest success, determined as the number of hatchlings successfully emerged from a clutch and quantified as a percentage, was arcsine transformed before being included in an LMM, again including δ^15^N, δ^13^C and CCL and interactions as fixed effects and with year and islands as random factors. Hatchling fitness was measured as individual size and mass as well as crawling and righting trials. LMMs for hatchling size and mass included parasite presence/absence, δ^15^N and δ^13^C, CCL, incubation duration and clutch size, and their two-way interactions, while crawling and self-righting trials also included hatchling mass.

## Results

### Clarifying leech taxonomy

Out of 90 randomly selected leeches, we successfully retrieved 67 and 86 sequences from18S rDNA and NADH respectively, representing all specimens (Supplementary Data File 2 and 3). All sequences confirmed turtles are infected with the *Ozobranchus margoi* leech—a sea turtle specific sanguivorous parasite^52^.

### Spatiotemporal trends in parasite prevalence

There was substantial variation in parasite prevalence among islands—infection was lowest in Fogo (FG, 1.33% ± 1.33 SE) and highest in Santiago (ST, 27.95% ± 9 SE). Parasite presence was significantly higher in turtles nesting on the eastern islands of Santiago (ST, 27.95% ± 9 SE), Boa Vista (BV 19.46% ± 5.47 SE), Maio (MA, 27.15% ± 5.70 SE) and Sal (SAL, 24.69% ± 8.34 SE) than those in the western region of the archipelago (São Nicolao, SN: 7.62% ± 7.61 SE, São Vicente, SV: 6.18% ± 2.85, Santo Antão, SA: 5.04% ± 2.74 SE, Santa Luzia, SL: 2.13% ± 0.91 SE, Fogo, FG: 1.33% ± 1.33 SE; Fig. 1B,C, X^2^ = 145.34, df = 1, *p* < 0.001). While a significant interaction between island and year suggested different island-specific trends in parasite prevalence in nesting turtles over time (Χ^2^ = 38.357, df = 8, *p* < 0.001), overall we detected an increase in the prevalence of *O. margoi* in turtles from Cabo Verde since 2010 (Fig. 1D), with multi-year oscillations. On average, infected turtles were slightly smaller than uninfected ones (Mean infected: 81.3 ± 0.39 SE cm, Mean uninfected: 82.45 ± 0.19 SE cm; Χ^2^ = 12.529, df = 1, *p* < 0.001). We also found evidence of within-year parasite dynamics, with infected turtles being significantly more likely to be encountered at the beginning of the nesting period than later in the season (Fig. 1E, Χ^2^ = 5.501, df = 1, *p* = 0.019; Model summaries in Supplementary Table S3).

### Impacts of infection on foraging strategy

**Figure 2:**
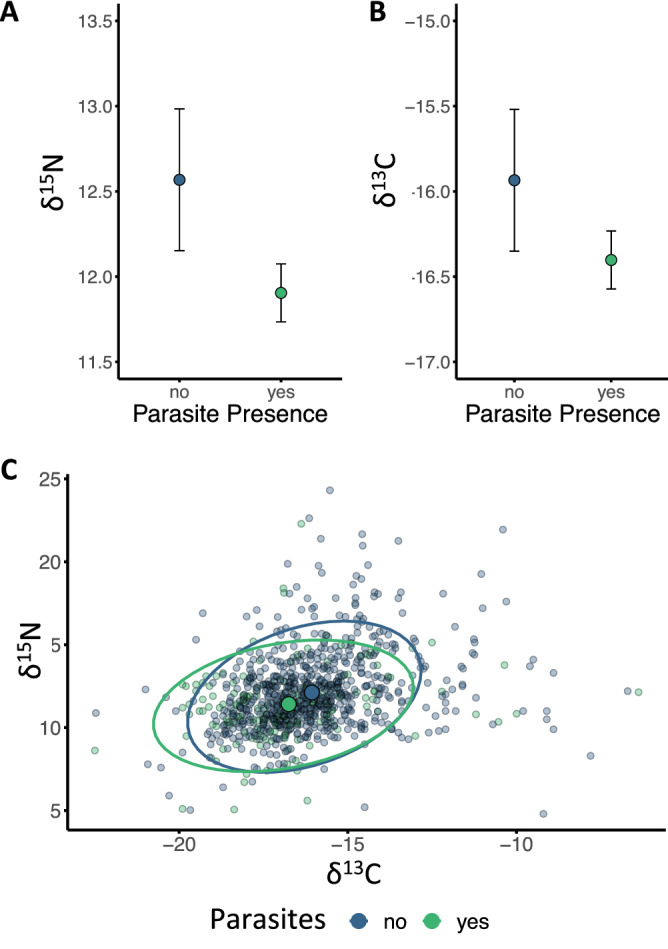
Infection correlates with differences in foraging strategy; (**A**) Mean δ^15^N values (F_1828_ = 9.551 p = 0.002) and (**B**) δ^13^C (F_1822_ = 7.562, *p* = 0.006) of infected and uninfected females (with standard error bars), as predicted from model outputs. (**C**) Difference in trophic niche of infected (green) and uninfected (blue) individuals, showing mean centroids and 95% confidence ellipses. Colours are consistent across panels.

Overall, there was a strong positive correlation between δ^15^N and δ^13^C values (Supplementary Fig. S1, F_1924_ = 84.603, *p* < 0.001), suggesting that turtles foraging at a lower trophic position, or in less δ^15^N enriched areas, forage in regions with a more depleted δ^13^C value. Turtle size, measured as CCL and controlled for island (F_8,4289_ = 39.933, *p* < 0.001), positively correlated with δ^15^N (F_1826_ = 10.865, *p* < 0.001) but not with δ^13^C (F_1823_ = 0.241, *p* = 0.624). We found that both the δ^15^N and δ^13^C were significantly lower in infected than uninfected individuals (Table 1, δ15N: Fig. 2A, F_1828_ = 9.551, *p* = 0.002; δ^13^C: Fig. 2B, F_1822_ = 7.562, *p* = 0.006). This amounted to a reduction of 0.66 ± 0.40 (SE) ‰ for δ^15^N and 0.47 ± 0.17 ‰ for δ^13^C. Overall, infected turtles occupied a slightly modified trophic niche in comparison to uninfected individuals (Fig. 2C).

**Table 1:** Best reduced models explaining the correlations of infection and CCL, along with their two-way interaction with (1) δ^15^N and (2) δ^13^C. All models were backwards selected using AIC.

	*d.f (numerator, denominator)*	F	*p*
**(1) δ**^**15**^**N**			
Parasite presence/absence	1828	9.551	**0.002**
CCL	1826	10.865	**0.001**
**(2) δ**^**13**^**C**			
Parasite presence/absence	1822	7.562	**0.006**
CCL	1823	0.241	0.624

Significant results highlighted in bold. D.f. denotes degrees of freedom.

### Impact of infection on reproductive output

**Figure 3:**
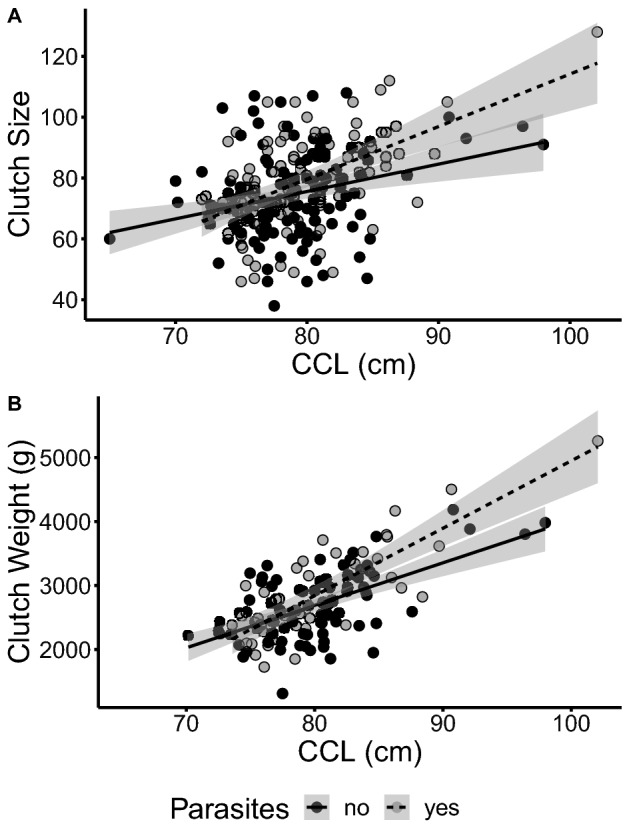
Infection interacts with the correlation between maternal size and both (**A**) clutch size (F_1128_ = 7.400, *p* = 0.007) and (**B**) mass (F_1110_ = 7.802, *p* = 0.006). Infected females produce larger clutches, with the effect being greatest at large size.

Carapace length (CCL) was the only maternal phenotype that correlated positively with the size and mass of individual eggs, with egg size increasing by 4.33 mm ± 0.58 SE with a 10 cm increase in maternal CCL, and egg mass increasing by 1.67 g ± 0.66 SE (Supplementary Fig. S2, size: F_199_ = 37.672, *p* < 0.001; mass: F_1119_ = 54.319, *p* < 0.001). We also found a significant interaction of maternal CCL and infection on both clutch size (Fig. 3A, F_1128_ = 7.400, *p* = 0.007) and overall clutch mass (Fig. 3B, F_1110_ = 7.802, *p* = 0.006). Specifically, small, infected females produced fewer eggs than their non-infected counterparts, while large infected turtles produced more eggs, which also resulted in greater clutch mass (Table 2). If we apply this measured effect size of infection on reproductive output to the clutch size and size structure of turtles nesting on Boa Vista, then infection results in a 1.21% net increase in reproductive output. Although δ^15^N and δ^13^C were not associated with any characteristics of reproductive investment, δ^15^N did show a positive relationship with the nest success of infected mothers (Supplementary Fig. S3, Table 3, F_1126_ = 10.731, *p* = 0.001). An interaction between maternal infection and CCL was also significantly correlated with nest success, whereby there was a positive correlation between CCL and success in uninfected turtles, but not infected turtles (F_1100_ = 9.361, *p* = 0.003).

**Table 2:** Best reduced models explaining the correlations of infection, CCL, δ^15^N and δ^13^C, along with their two-way interactions, with reproductive investment, including; (1) average egg size, (2) average egg mass, (3) clutch size, (4) clutch mass and (5) nest success. All models were backward selected using AIC criteria.

	*d.f (numerator, denominator)*	F	*p*
**(1) Average egg size**			
Parasite presence/absence	1113	0.380	0.534
CCL	199	37.672	** < 0.001**
δ^13^C	172	0.465	0.497
Parasite presence: δ^13^C	1112	2.027	0.157
**(2) Average egg mass**			
CCL	1119	54.319	** < 0.001**
**(3) Clutch size**			
Parasite presence/absence	1127	3.799	0.053
CCL	1127	0.589	0.444
δ^15^N	1107	0.100	0.752
Parasite presence: CCL	1128	7.400	**0.007**
CCL: δ^15^N	1127	2.371	0.126
**(4) Clutch mass**			
Parasite presence/absence	1113	3.873	0.051
CCL	1112	103.936	** < 0.001**
δ^15^N	121	2.625	0.119
Parasite presence: CCL	1110	7.802	**0.006**
**(5) Nest success**			
Parasite presence/absence	1125	0.243	0.623
CCL	1115	0.178	0.674
δ^15^N	1122	1.924	0.168
Parasite presence: CCL	1100	9.361	**0.003**
Parasite presence: δ^15^N	1126	10.731	**0.001**
CCL: δ^15^N	1126	3.891	0.051

Significant results highlighted in bold. d.f. denotes degrees of freedom.

### Transgenerational impact of maternal infection on offspring fitness

An interaction between maternal infection and clutch size was significantly associated with offspring SCL (Table 3, Supplementary Fig. S4A, F_1226_ = 6.921, *p* = 0.009): hatchlings originating from small clutches produced by infected females were smaller than those produced by uninfected females, while the opposite relationship was detected in large clutches, with hatchlings from infected females being bigger. This effect did not remain in the reduced model of hatchling mass. The interaction between maternal infection and clutch size also significantly correlated with self-righting speed (Supplementary Fig. S4B, F_1114_ = 8.413, *p* = 0.004), in which offspring from infected mothers righted themselves on average 17% faster than offspring from uninfected mothers (average self-righting speed of offspring from infected mothers: 6.51 ± 0.22 (SE) seconds; uninfected mothers: 7.84 ± 0.19 (SE) seconds), but this difference was strongest when clutch sizes were small. The same interaction between maternal infection and clutch size was detected when investigating self-righting success (X^2^ = 3.681, df = 1, *p* = 0.055), but not when testing crawl speed (F_1111_ = 2.939, *p* = 0.089).

**Table 3:** Best reduced models explaining the correlation of infection, CCL, δ^15^N and δ^13^C, incubation duration and clutch size along with their two-way interactions, with hatchling phenotype including (1) size and (2) mass. All models were backward selected using AIC criteria.

	*d.f (numerator, denominator)*	F	*p*
**(1) Hatchling size**			
Parasite presence	1225	3.416	0.066
CCL	1231	4.497	**0.035**
Incubation duration	1232	6.121	**0.014**
Cutch size	1225	19.292	** < 0.001**
Parasite presence: clutch size	1226	6.921	**0.009**
CCL: incubation duration	1232	5.769	**0.017**
**(2) Hatchling mass**			
CCL	1313	29.904	** < 0.001**
Incubation duration	1208	5.955	**0.016**
Clutch size	1223	7.499	**0.007**

Significant results highlighted in bold. D.f. denotes degrees of freedom.

An interaction between hatchling mass and maternal δ^15^N was significantly associated with both self-righting (Supplementary Table 4, Supplementary Fig. S5, F_12525_ = 5.163, *p* = 0.023) and crawl speeds (Supplementary Fig. S5, F_12458_ = 4.993, *p* = 0.026). Offspring from females with a higher δ^15^N were faster in both tests, although this was dependent on the mass of the hatchlings, with the effect being greatest in the heaviest ones.

## Discussion

Long-term monitoring of endangered species improves the understanding of population dynamics and responses to environmental change. In the current study, we found that over eight years and across 4300 sea turtle samples, the prevalence of infection by the sanguivorous leech *Ozobranchus margoi* in Cabo Verde has increased, from 10% of the population being infected in 2010, to 33% in 2017. Infected individuals had more depleted δ^13^C stable isotope ratios, which are indicative of oceanic feeding and suggests elevated transmission within oceanic foraging grounds or among oceanic turtles^48,53^. Furthermore, infected turtles showed significantly lower δ^15^N values, suggesting a relationship between their feeding ecology and parasite infection. Interestingly, the link between infection and reproduction is female size-dependent. Large infected turtles produced more eggs per clutch with bigger offspring than their uninfected counterparts, while smaller infected individuals produced fewer eggs and smaller hatchlings. Offspring from all infected turtles performed better in self-righting tests, providing evidence of positive maternal effects associated with infection. Using size as a broad proxy for age, we uphold the suggestion that the cost of infection could be borne differently across life stages. We propose that while small/young infected turtles could use a bet-hedging strategy in favour of lifetime reproductive success, older infected turtles adopt a terminal investment strategy. Noteworthy, these coexisting strategies result in a slight (1.21%) net increase in the reproductive output of this population compared to a theoretical turtle population without parasites. This slight advantage is sufficient for the proposed reproductive strategies to evolve in the population in response to parasite infection and compensate for the otherwise costly infection at the individual level.

The sanguivorous leech, *O. margoi,* can infect most, if not all, sea turtle species^20^. Since 2010, *O. margoi* prevalence in the Cabo Verde loggerhead sea turtle population has increased, and shows the classic oscillations of parasite infection^54^. The fact that the timing of oscillations is similar across islands implies that infection occurs outside the nesting grounds, rather than within island-specific breeding groups^44^. These multiyear oscillations are likely caused by a complex interaction between environmental factors and host-parasite dynamics, as would be predicted by the Red Queen hypothesis^54–56^. Alternatively, as female turtles nest every two to three years, these oscillations could stem from a cohort effect. However, we consider this explanation less likely because nesting return rates vary for each individual.

Our results also suggest that parasite prevalence is not homogenous across islands, as the prevalence of infection is significantly higher in the east of the archipelago, where nesting densities are higher^44^. The existence of island-specific variation in immune genes of the major histocompatibility complex within this sea turtle population could explain differential levels of local adaptation in the host and hence this distribution^44^. Alternatively, it could be that some density dependent transmission is maintained in large nesting groups, for instance, during mating. Increasing prevalence of this parasite implies an increased risk from the ChHV5 virus, responsible for fibropapillomatosis^57^. This virus has now been recorded in all species and all ocean basins^19,57^. If it reaches Cabo Verde, it could pose a considerable threat to this already vulnerable population.

When investigating how parasite infection correlates with feeding ecology, we found a relationship between parasite presence and the isotopic values of δ^15^N and δ^13^C. Two hypotheses exist to explain the relationship with δ^15^N: (1) turtles foraging at lower trophic levels exploit niches which make them more prone to be exposed to and infected by the parasites (e.g. in brook charr, *Salvenlinus fontinalis*^58^) or (2) infection reduces health, and trade-offs with foraging efficiency cause turtles to target more prey items from lower trophic positions, which are probably easier to catch and of lower nutritional value than prey of uninfected turtles^59,60^. Outside the breeding season, before their migration to nesting grounds, most turtles from Cabo Verde forage in oceanic habitats, either opportunistically on neustonic organisms such as jellyfish, or at higher nitrogen levels in regions exposed to upwelling events^48,61^. A much smaller proportion forage in neritic waters^48,62^. As more depleted δ^13^C ratios are indicative of open-ocean foraging, the depleted ratios of infected turtles may suggest that transmission of *O. margoi* occurs at a higher rate in oceanic feeding grounds or among oceanic foragers, or even that oceanic foragers are more susceptible to infection^48,53^. If infection is linked to oceanic foraging, infection of neritic turtles could occur at lower rates in the coastal habitats during mating. As there is no evidence that turtles with different foraging strategies segregate during mating^44^, if substantial transmission occurred at this time, we would expect to see no difference in δ^13^C ratio between infected and uninfected individuals.

From an evolutionary perspective, infection may translate into altered lifetime reproductive success and ultimately impact fitness^30^. Infected turtles that forage at enriched δ^15^N levels (associated with productive upwelling regions) are on average larger, and produce clutches that have a greater rate of success than those infected individuals with a lower δ^15^N. High levels of productivity within upwelling regions are likely to allow turtles to successfully forage and compensate from the costs of infection and mounting energetically costly immune responses^63,64^.

The δ^15^N and δ^13^C ratios of individuals did not show a relationship with any characteristics of reproductive investment. Instead, we found that the relationship between maternal size and clutch size was influenced by the presence of parasites. Small, infected turtles produced fewer eggs per clutch than their uninfected equivalents. In turn, smaller and lighter offspring hatched from those clutches. This relationship was reversed in large infected turtles, which produced bigger clutches and heavier offspring than uninfected conspecifics. As sea turtles grow continuously throughout their life, we may expect that on average, larger sea turtles are older^65^. Our results point at the coexistence of two size/age specific reproductive strategies. Small, i.e. probable young, turtles might follow a bet-hedging strategy, whereby if infected, they reduce investment, to reserve resources for future reproductive events^64^. On the other hand, large, presumably older, turtles terminally invest, with infected individuals maximising their current reproductive success as there may be few, if any, reproductive events in the future^66^.

As for all field studies on host-parasite interactions, it is impossible to determine the direction of causality between host health and infection. But, if unhealthy turtles are more susceptible to infection, then poor general fitness could be an alternative explanation for the lower reproductive output of small, infected turtles. However, this hypothesis would not explain elevated reproductive investment in large infected individuals. Little is known about the interaction between leech infection and host health in this system^39,40^. Such studies will form the next steps of research to understand the underlying mechanisms of the relationships we have reported.

Regardless of the underlying mechanism, the relationships presented here reveal proxies that link infection and reproductive success. If we apply the observed relationship between adult size and clutch size for these two reproductive strategies to the size class structure in Boa Vista, we observe a small, 1.21%, net increase in reproductive output of this population. This is in contrast to the strong negative effect of parasites at the population level that has frequently been observed in bird and mammal populations for instance^9^. Our results highlight the evolutionary role of host-parasite dynamics, which leads to the evolution of strategies that maximise population reproductive success.

Offspring size and self-righting ability correlated with an interaction between maternal infection and clutch size. In addition, hatchlings from all infected females performed on average 17% faster in self-righting tests than offspring from uninfected mothers. While this difference was not mirrored in the crawl tests, it provides some evidence that trans-generational maternal effects confer fitness benefits that may contribute to dispersal^67^. Particularly, combined with the negative correlation between self-righting time and offspring mass, the maternal effects of large females may maximize dispersal capacity as larger offspring have better swimming capacity than smaller ones^49^. An elevated body condition enables offspring to access currents that propel them away from predatory rich coastal areas^49^. Other variables, such as the thermal and hydric conditions of the nests, also affect the physiology and locomotor skills of sea turtle hatchlings^50,68^. While we did not systematically quantify abiotic conditions, we minimised abiotic variation by relocating nests to a single hatchery on each island, exposing the nests to similar substrates, moisture content and temperatures. Even if such external variables were to correlate with trans-generational effects, our study reveals infection as a simple proxy to monitor those effects.

Long-term field monitoring projects will provide the first evidence of changing host-parasite dynamics within a population. Such studies are therefore crucial for effective conservation management. It is evident that *O. margoi* infection is costly to loggerhead sea turtle hosts, which have evolved different reproductive strategies based on their size. Interestingly, by combining these effects with the size class structure of the island of Boa Vista, we observe a net increase in reproductive output of 1.21% in response to *O. margoi* infection, illustrating how monitoring the effects of an infection aids our understanding of population demographics. Future studies should (1) determine the cause of the increase in parasite prevalence; (2) determine whether it is host density or environmentally mediated and (3) explore the relationship between turtle health and infection. This study provides the necessary basis for these research avenues.

## Supplementary information

Supplementary Information 1.

Supplementary Information 2.

Supplementary Information 3.

Supplementary Information 4.
