# Supplementary material for: Long-term survey of sea turtles (Caretta caretta) reveals correlations between parasite infection, feeding ecology, reproductive success and population dynamics
Source: Sci Rep. 2020 Oct 29;10:18569. doi: 10.1038/s41598-020-75498-4 (PMC7596700; doi:10.1038/s41598-020-75498-4)
Supplement: Supplementary file 4 — Supplementary Information 4. [file 41598_2020_75498_MOESM4_ESM.pdf]

Supplementary Material for: Long-term survey of sea turtles (*Caretta caretta*) reveals correlations between parasite infection, feeding ecology, reproductive success and population dynamics.

Emma C. Lockley[1*], Leila Fouda[1], Sandra M. Correia[2], Albert Taxonera[1,3], Liam N. Nash[1], Kirsten Fairweather[3], Thomas Reischig[4], Jandira Durão[5], Herculano Dinis[6], Silvana Monteiro Roque[7], João Pina Lomba[8], Leno dos Passos[9], Sahmorie J. K. Cameron[1], Victor A. Stiebens[1], Christophe Eizaguirre[1]

Table S1: Composition of 20 µl PCR reactions for NADH and 18S rDNA. All PCR reactions were carried out under the same conditions: Thermo-cycling began with initial denaturation at 94 °C for 4 minutes followed by 45 cycles at 94 °C for 2 minutes 15 seconds, 44 °C for 20 seconds, 70 °C for 1 minute 30 seconds. The final extension lasted 7 minutes at 70 °C.

**Constituent**	**Volume**
Taq Polymerase (Biosystem Red Mix, 2x)	10 µl
F and R primer (5pmol/µl)	2 µl each
HPLC water	2 µl
template DNA	4 µl

Table S2: List of models tested. All two-way interactions between fixed effects were also included in full models.

Model	Response	Fixed Effects	Random Effects	Model Type
	Spatiotemporal trends			
1	Parasite Presence/Absence	Year, Island, CCL	NA	Generalised linear model (binomial)
2	Parasite Presence/Absence	Month, Island, CCL	Year	Generalised linear mixed effects model (binomial)
	Foraging Strategy			
3	$\delta^{15}N$	Parasite presence/absence, CCL	Island, Year	Linear mixed effects model
4	$\delta^{13}C$	Parasite presence/absence, CCL	Island, Year	Linear mixed effects model
	Reproductive Investment			
5	Average Egg Mass	Parasite presence/absence, CCL, $\delta^{15}N$, $\delta^{13}C$	Island, Year	Linear mixed effects model
6	Average Egg Size	Parasite presence/absence, CCL, $\delta^{15}N$, $\delta^{13}C$	Island, Year	Linear mixed effects model
7	Clutch Size	Parasite presence/absence, CCL, $\delta^{15}N$, $\delta^{13}C$	Island, Year	Linear mixed effects model
8	Clutch Mass	Parasite presence/absence, CCL, $\delta^{15}N$, $\delta^{13}C$	Island, Year	Linear mixed effects model
9	Success Rate	Parasite presence/absence, CCL, $\delta^{15}N$, $\delta^{13}C$	Island, Year	Generalised linear mixed effects model (binomial)
	Trans-generational Effect			
10	SCL	Parasite presence/absence, CCL, Clutch Size, Incubation Duration, $\delta^{15}N$, $\delta^{13}C$	Nest, Island, Year	Linear mixed effects model
11	Mass	Parasite presence/absence, CCL, Clutch Size, Incubation Duration, $\delta^{15}N$, $\delta^{13}C$	Nest, Island, Year	Linear mixed effects model
12	Run Time	Parasite presence/absence, CCL, Clutch Size, Incubation Duration, $\delta^{15}N$, $\delta^{13}C$, hatchling Mass	Nest, Island, Year	Linear mixed effects model
13	Flip Time	Parasite presence/absence, CCL, Clutch Size, Incubation Duration, $\delta^{15}N$, $\delta^{13}C$, hatchling Mass	Nest, Island, Year	Linear mixed effects model
14	Flip Success Rate	Parasite presence/absence, CCL, Clutch Size, Incubation Duration, $\delta^{15}N$, $\delta^{13}C$, hatchling Mass	Nest, Island, Year	Generalised linear mixed effects model (poisson)

Table S3: Statistical summary table reporting the best reduced models associated with parasite prevalence over 1) year and 2) season. All models were backwards selected using AIC. Significant results highlighted in bold. D.f. denotes degrees of freedom.

**1) Infection across years**	***d.f.***	**Chi-sq**	**p**
Year	1	194.669	**<0.001**
Island	8	102.237	**<0.001**
CCL	1	12.529	**<0.001**
Year:Island	8	38.357	**<0.001**
**2) Infection across season**			
Month	1	5.501	**0.019**
CCL	1	14.191	**<0.001**
Island	8	63.038	**<0.001**

Table S4: Statistical summary table reporting the best reduced models associated with maternal infection status (presence/absence of parasites), CCL, $\delta^{15}N$ and $\delta^{13}C$, incubation duration, clutch size and hatchling mass, along with their two-way interactions, on offspring fitness tests including 1) Self-righting success 2) self-righting speed and 3) crawl speed. All models were backwards selected using AIC. Significant results highlighted in bold. D.f. denotes degrees of freedom.

**1) Self-righting success**	***d.f.***	**X^2**	**p**
Parasite Presence	1	0.526	0.468
CCL	1	2.2699	0.132
**Incubation Duration**	**1**	**4.77**	**0.029**
Clutch Size	1	1.312	0.252
Hatchling Mass	1	2.918	0.088
$\delta^{15}N$	1	0.007	0.935
**Parasite Presence:CCL**	**1**	**5.3002**	**0.021**
Parasite Presence:Clutch Size	1	3.681	0.055
**CCL:Incubation Duration**	**1**	**4.038**	**0.044**
**CCL: $\delta^{15}N$**	**1**	**7.327**	**0.007**
Incubation Duration:Clutch Size	1	3.435	0.064

**2) Self-righting speed**	***d.f.***	**F**	**p**
**Parasite Presence**	**1,115**	**7.087**	**0.009**
CCL	1,119	0.015	0.903
Incubation Duration	1,116	1.496	0.224
Clutch size	1,115	0.031	0.859
**Hatchling Mass**	**1, 2526**	**4.883**	**0.027**
$\delta^{15}N$	1,116	2.014	0.159
$\delta^{13}C$	1,113	0.582	0.447
Parasite Presence:CCL	1,119	2.298	0.132
**Parasite Presence:Clutch Size**	**1,114**	**8.413**	**0.004**
Parasite Presence: $\delta^{13}C$	1,114	3.819	0.053
CCL:Clutch Size	1,115	2.742	0.100
**Incubation Duration:Clutch Size**	**1,114**	**4.416**	**0.038**
**Hatchling Mass: $\delta^{15}N$**	**1, 2525**	**5.163**	**0.023**
Hatchling Mass: $\delta^{13}C$	1, 2526	3.389	0.066

**3) Crawl Speed**			
Parasite Presence	1,111	0.981	0.324
CCL	1,119	2.185	0.142
Incubation Duration	1,103	0.353	0.554
Clutch Size	1,110	0.011	0.918
**Hatchling Mass**	**1, 2458**	**5.003**	**0.025**
$\delta^{15}N$	1,75	3.295	0.073

$\delta^{13}C$	1,110	0.6799	0.411
Parasite Presence:Clutch Size	1,111	2.939	0.089
Parasite Presence: $\delta^{15}N$	1,112	2.688	0.104
Parasite Presence: $\delta^{13}C$	1,110	3.813	0.053
CCL:Clutch Size	1,110	2.308	0.131
**CCL:** $\delta^{15}N$	**1,167**	**8.323**	**0.004**
**Incubation Duration:Clutch Size**	**1,111**	**5.284**	**0.023**
Clutch Size: $\delta^{13}C$	1,110	2.4296	0.122
**Hatchling Mass:** $\delta^{15}N$	**1, 2458**	**4.993**	**0.026**
$\delta^{15}N$: $\delta^{13}C$	1,109	3.29	0.072

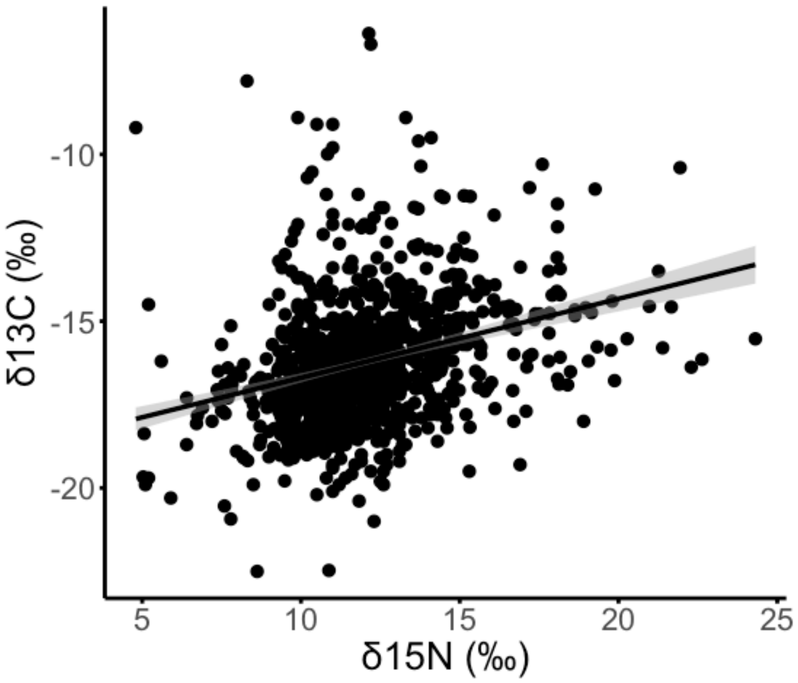

Figure S1: Positive correlation between $\delta^{15}N$ and $\delta^{13}C$ values of skin samples from adult nesting females ($F_{1,924} = 84.603$, $p < 0.001$).

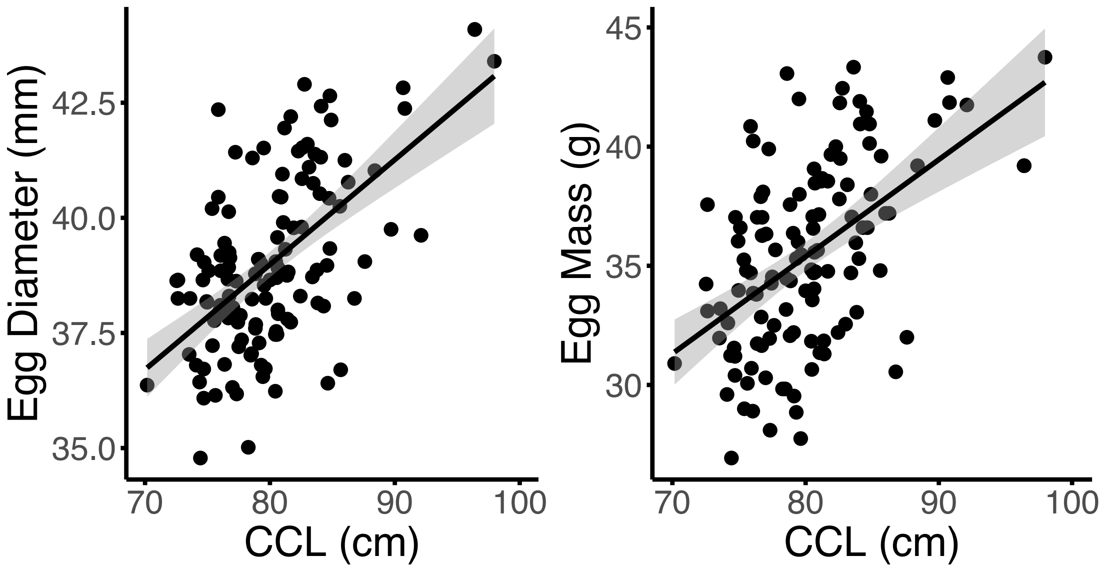

Figure S2: Maternal size significantly correlated with egg diameter ($F_{1,99}$ = 37.672, p < 0.001) and egg mass ($F_{1,119}$ = 54.319, p < 0.001).

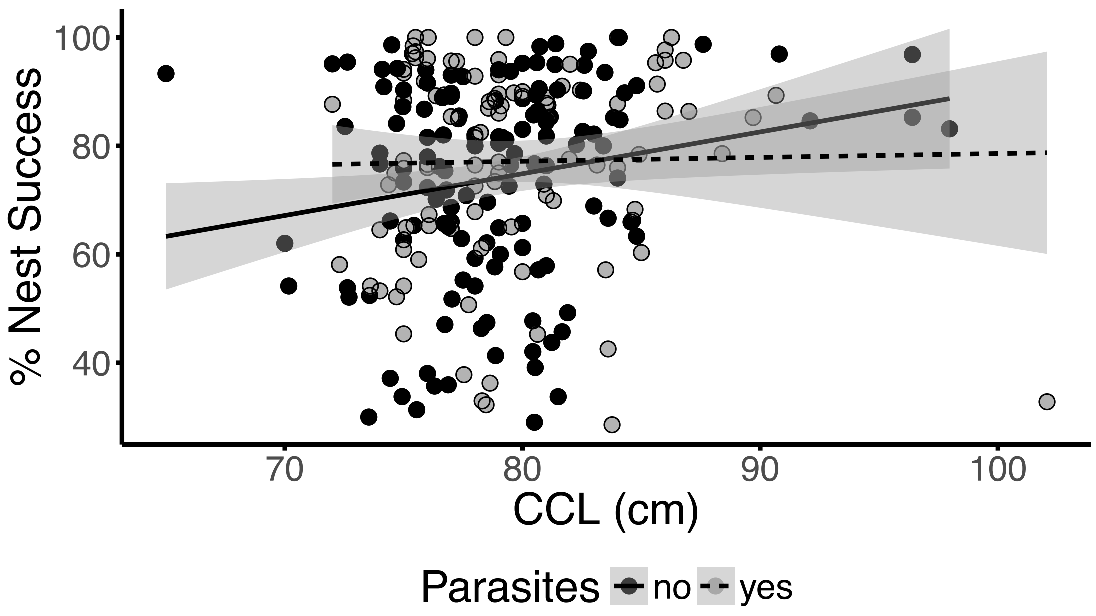

Figure S3: Interaction effect between the $\delta^{15}N$ values and infection status of a nesting female turtle on the success rate of a nest ($F_{1,126} = 10.731$, $p = 0.001$).

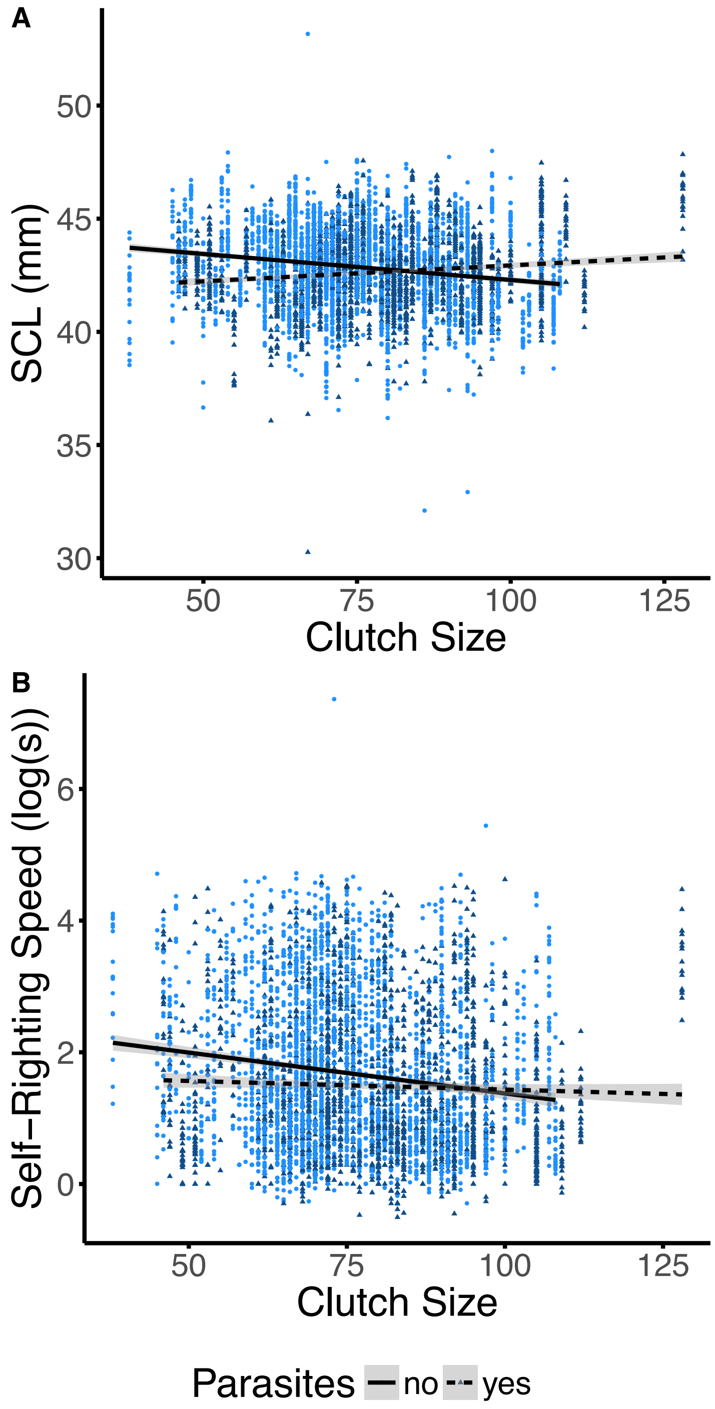

Fig S4: The interaction between maternal infection status and clutch size was significantly associated with both hatchling size ($F_{1,226}$ = 6.921, p = 0.009) and self-righting speed ($F_{1,114}$ = 8.413, p < 0.004)

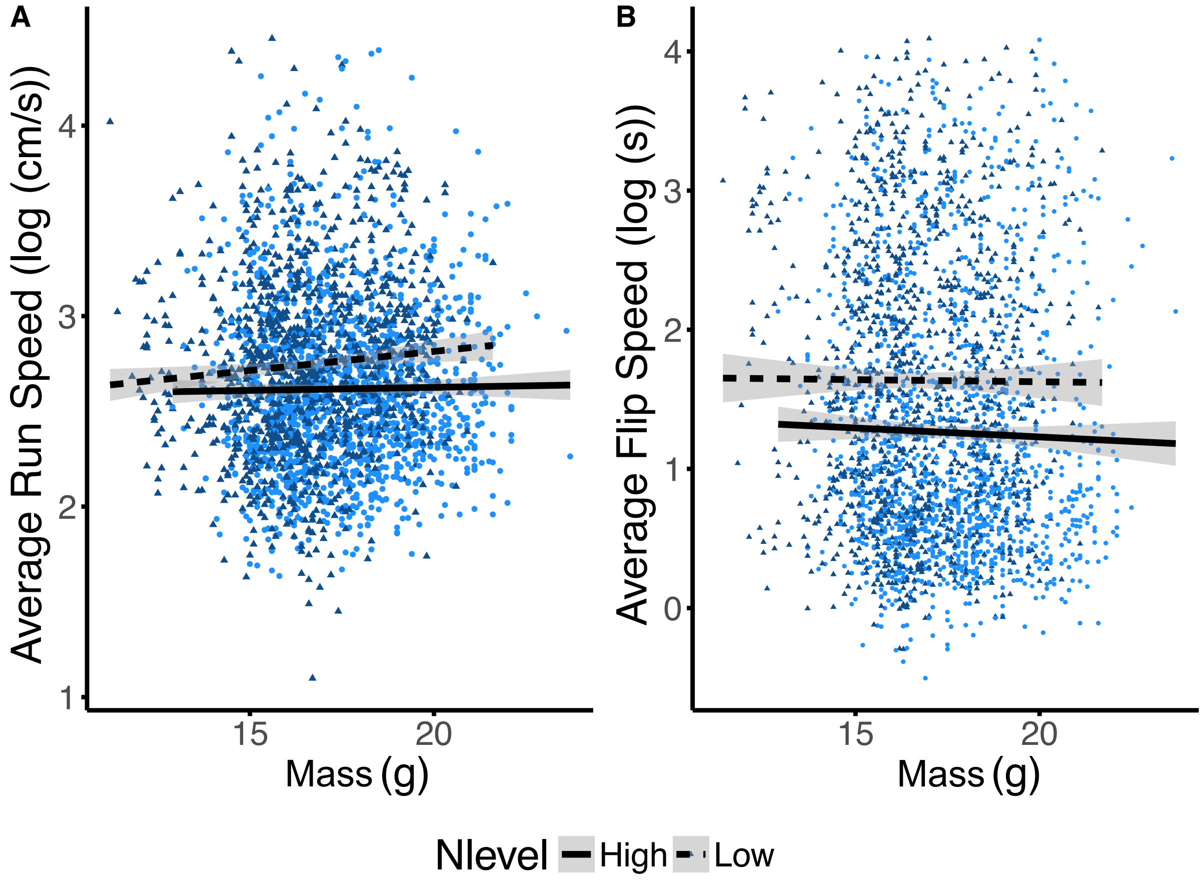

Fig S5: A significant interaction was detected between hatchling mass and the $\delta^{15}N$ of its mother on the speed it performs both crawl ($F_{1,2458}$ = 4.993, p = 0.026) and self-righting tests ($F_{1,2525}$ = 5.163, p = 0.023). Individuals from mothers that foraged at an enriched $\delta^{15}N$ trophic position performed faster in both tests, with this effect being greatest in the heaviest hatchlings..
